# Metastases from gastric cancer presenting as colorectal lesions: a report of two cases and systematic review

**DOI:** 10.1308/rcsann.2023.0023

**Published:** 2023-05-23

**Authors:** VL Fretwell, EG Kane, S MacPherson, P Skaife

**Affiliations:** Liverpool University Hospitals NHS Foundation Trust, UK

**Keywords:** Gastric cancer, Colorectal cancer, Metastasis

## Abstract

Gastric cancer is common with well-established routes of spread. Metastasis to the colon or rectum is rare; however, we have recently managed two patients with this clinical picture. We present these cases together with a literature review of current practice. A systematic review in PubMed using the terms ‘gastric cancer’ and ‘colorectal metastasis’ was performed. The identified papers were screened for relevance and the reference lists of relevant papers were also reviewed to ensure capture of all relevant reports. Twenty-four papers containing 26 cases of gastric cancer with metastasis to the colon or rectum were found. There was wide variation in presentation and practice in these cases, which tended to be in patients with poor histopathological features. Diagnosis is often challenging owing to the unusual radiological appearance and submucosal nature of the metastatic lesions. Treatment ranges from palliative care to radical resection. Colorectal metastases from gastric primary cancer are rare, but cases are reported and should be part of the index of suspicion during the work-up of patients with lower gastrointestinal symptoms and a history of gastric cancer. Treatment options range from aggressive surgical resection to palliative care and should be centred on the patient’s fitness and wishes.

## Background

Gastric cancer is common. Worldwide there were an estimated 1.1 million new diagnoses of gastric cancer in 2020, with the highest incidence and mortality rates in East Asia.^1^ In the UK approximately 6,500 people are diagnosed annually.^2^ National Institute for Health and Care Excellence (NICE) guidelines recommend pre- and postoperative chemotherapy in surgically resectable cases, and palliative chemotherapy with or without trastuzumab is recommended in inoperable cases.^3^ Metastatic disease usually presents in the liver, lymph nodes and peritoneum^4^ via haematogenous or lymphangiomatous spread or via direct invasion into adjacent structures.

Metastasis of gastric cancer to the colon and rectum is rare but has been reported in the literature.^5^ Management of these lesions is highly variable. We present two cases we have managed in our unit and a systematic review of the current literature.

## Systematic literature review

A literature review was performed in PubMed on 16 October 2022. The search terms used were ‘gastric cancer’ AND ‘colorectal metastasis’. Papers were screened for relevance and excluded accordingly. Foreign language papers were excluded. The reference lists from the selected papers were interrogated for further relevant articles and further exclusions were made as detailed in the accompanying PRISMA diagram.

## Case histories

### Case 1

**Figure 1:**
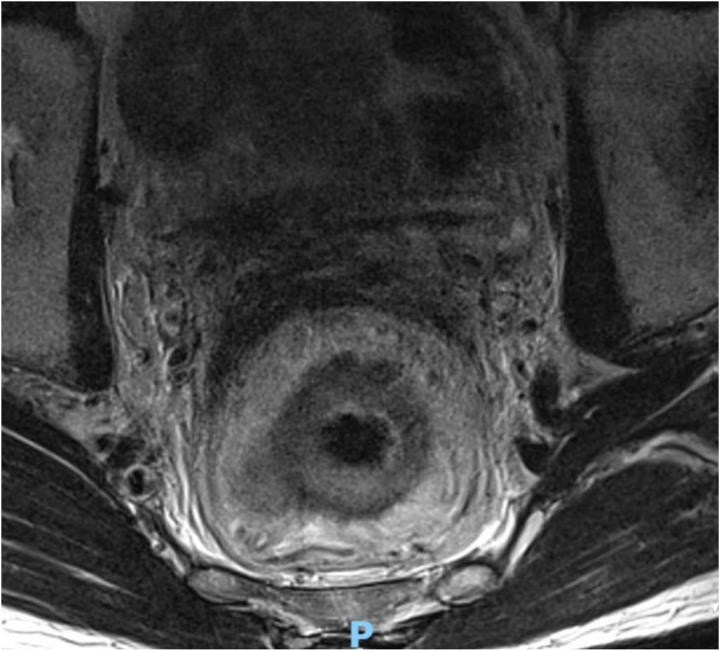
Magnetic resonance image of the rectum demonstrating annular thickening of the mid to upper rectum starting 7.5cm above the anal verge with linear T2 signal suggesting mucinous component

**Figure 2:**
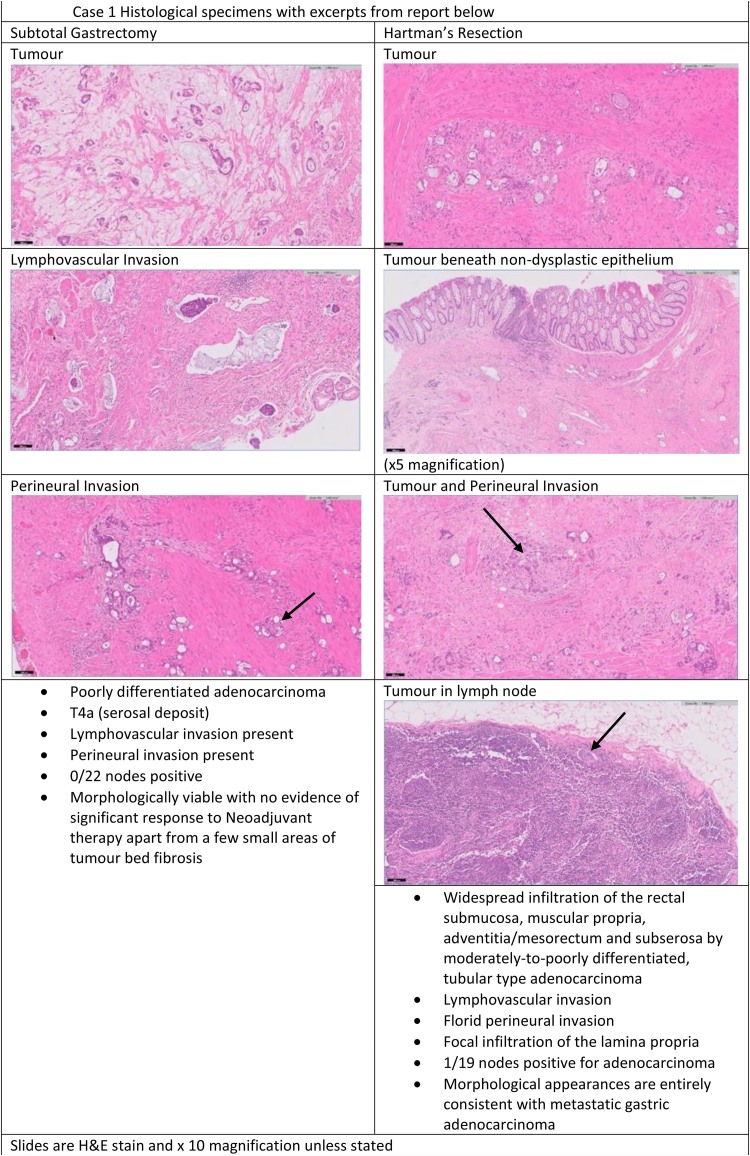
Histological specimens from Case 1

A 65-year-old man with a World Health Organization (WHO) performance status of 0 was investigated in April 2016 for weight loss, nausea and decreased appetite. Gastroscopy showed an abnormality in the antrum of the stomach and biopsies showed a moderately differentiated adenocarcinoma. Computed tomography (CT) and endoscopic ultrasound imaging staged the disease as T3/4aNM0. The patient was given a trial of neoadjuvant chemotherapy that was halted after two cycles owing to side effects. A subtotal gastrectomy with radical lymphadenectomy was performed in September 2016 with final histological staging of a poorly differentiated adenocarcinoma T4aN0R0 (0/22 lymph nodes involved). Adjuvant chemotherapy was not given in view of previous poor tolerance and limited impact on tumour stage. The patient presented 20 months after his gastric surgery with rectal pain and tenesmus. Magnetic resonance imaging (MRI) of the rectum demonstrated annular thickening of the mid to upper rectum starting 7.5cm above the anal verge, with minor stranding of the serosa and mesorectal fat but no significant lymphadenopathy (Figure 1). An examination under anaesthetic of the rectum demonstrated an ulcerated area which was biopsied, showing inflammatory change only. The patient had a defunctioning colostomy formed to manage his symptoms. Two months later owing to ongoing symptoms and clinical concern, the patient underwent an ultra-low Hartmann’s procedure. The operative findings were of peritoneal nodules and an advanced, fibrotic, ‘woody’ feeling rectal tumour, tight in the pelvis with compression of the pelvic side walls and extending further distally than imaging had suggested, leading to a challenging dissection. Histology from the rectum demonstrated metastatic gastric adenocarcinoma in the rectum and pericolonic fat, which was present at the distal resection margin and circumferential resection margin (Figure 2). The patient had two admissions with small bowel obstruction managed conservatively, but was never well enough for any adjuvant chemotherapy. He died 6 months following his rectal surgery.

### Case 2

**Figure 3:**
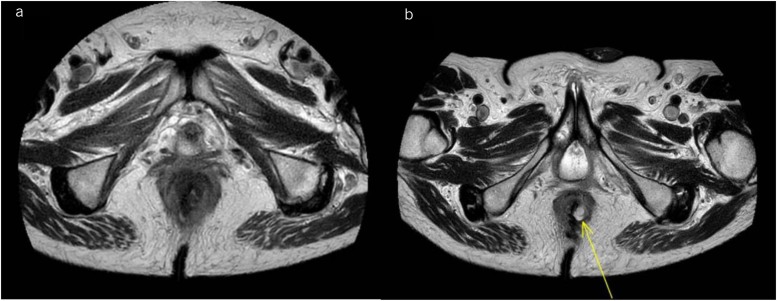
(a) Diffuse circumferential thickening involving the anal canal and entire rectum with surrounding inflammation in mesorectal fat and multiple tiny mesorectal lymph nodes. (b) A 12mm high T2 signal in left posterior anal canal suspicious for small collection (yellow arrow). Overall appearances favour ano-proctitis; however, underlying malignancy cannot be excluded.

**Figure 4:**
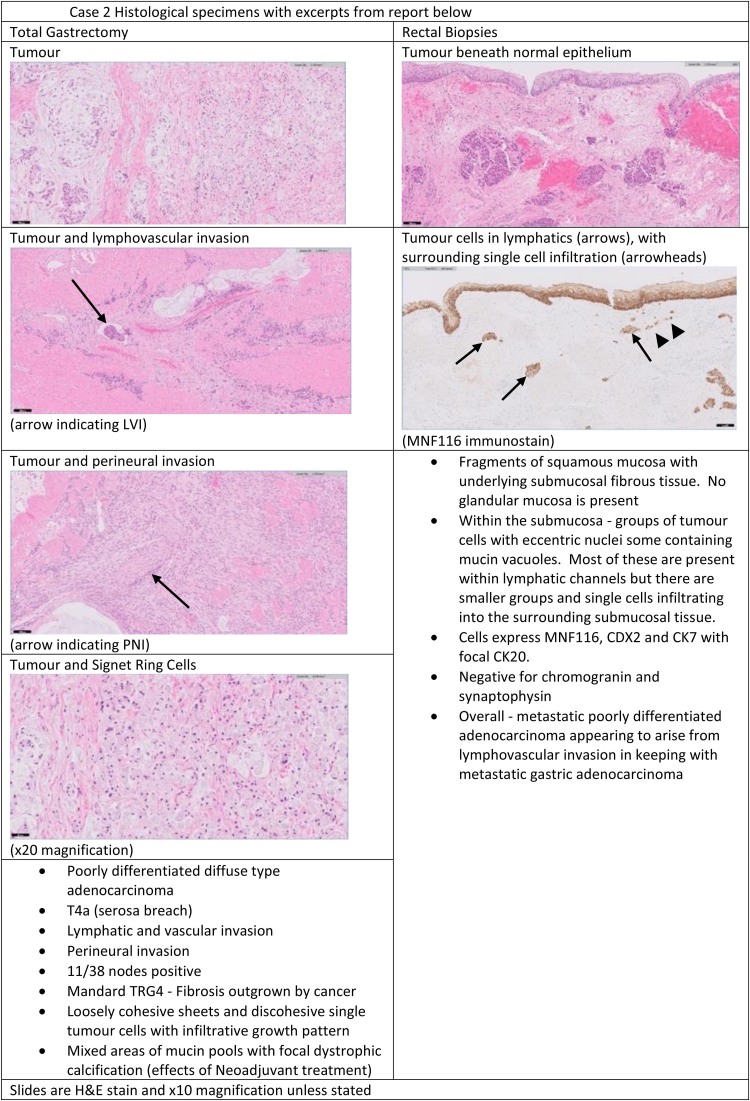
Histological specimens from case 2

A 61-year-old man with a WHO performance status of 0–1 presented with dysphagia, anaemia and weight loss in October 2020. A gastroscopy showed a large malignant antral polypoidal mass with central ulceration. Biopsies revealed poorly differentiated adenocarcinoma with signet cell foci. A full-staging CT scan staged the disease as T4N1aM0. The patient underwent neoadjuvant chemotherapy between December 2020 and March 2021. In April 2021, he had a D2 total gastrectomy (T4aN3aR0 11/38 nodes) it having been noted intraoperatively that the tumour was advanced, with poor response to neoadjuvant treatment, requiring total gastrectomy. The patient made a straightforward recovery followed by adjuvant chemotherapy. Seven months later he presented to his general practitioner with right-sided abdominal pain and a CT scan showed hydronephrosis and bilateral pyelonephritis. There was a palpable nodularity on rectal examination, so MRI rectum was performed showing diffuse circumferential rectal thickening involving the anal canal and entire rectum with surrounding inflammatory stranding and multiple tiny lymph nodes in the mesorectum (Figure 3). His carcinoembryonic antigen (CEA) level was normal at 1.8. An examination under anaesthetic of the rectum demonstrated a smooth tight anal stenosis with nodularity beneath the mucosa circumferentially. Biopsy showed metastatic poorly differentiated adenocarcinoma that appeared to arise from lymphovascular invasion suggestive of gastric origin (Figure 4). Despite being referred for palliative chemotherapy, the patient died 2 months later following hospice-led end-of-life care.

## Literature review

**Figure 5:**
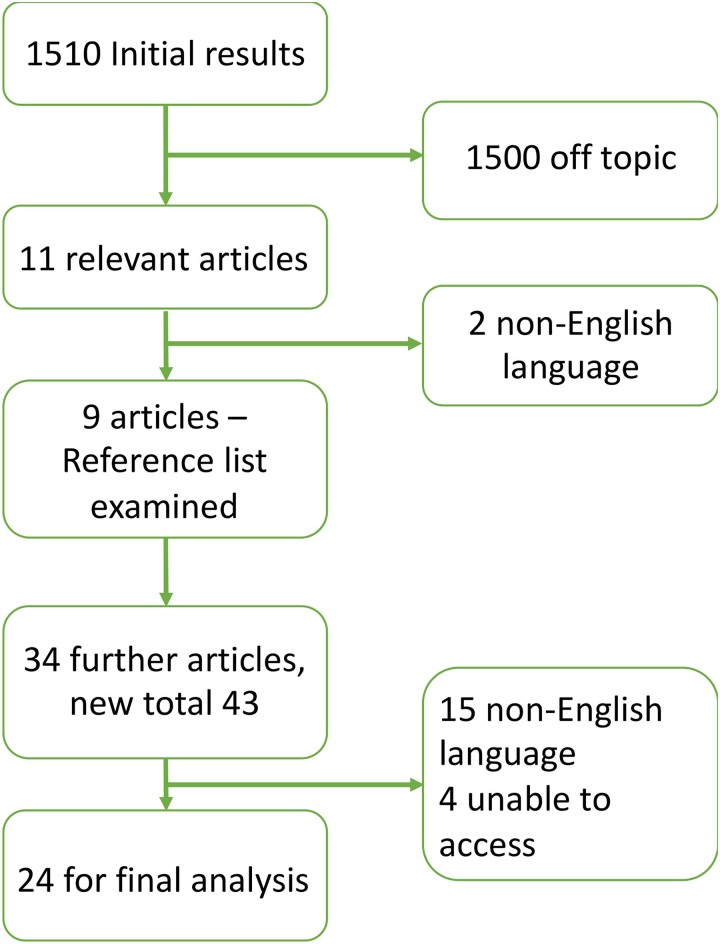
PRISMA diagram of the search strategy including inclusion and exclusions

The literature search identified 24 papers containing 26 cases of gastric cancer metastasising to the colon or rectum. The results of the search strategy are presented in the PRISMA diagram (Figure 5). The key presenting features and management approaches are given in Table 1.

**Table 1:** Summary of key points from the case reports

Paper	Age/sex	Gastric surgery	Histology (gastric)	TNM (postoperative)	Lower gastrointestinal presentation	Colorectal findings	Tumour markers	Management of colorectal lesion	Time to metastasis (months)	Overall survival (months)
Wang *et al* 2021^6^	42 M	Curative resection	PDA	T3N2M0	Diarrhoea + weight loss	Stenotic lesion 60cm from anal verge and polypoid lesion in sigmoid	Normal	Colonic and peritoneal resection 2 further colonic resections subsequently	110	154
Yang *et al* 2019^7^	57 M	Curative resection	PDA + SR	T3N2M0	RIF pain	Annular stricture in right colon involving ileocaecal valve	Raised	Right hemicolectomy and lymphadenectomy. Adjuvant chemoradiation	30	39
Su *et al* 2018^8^	77 M	None. Synchronous metastases	PDA	N/A	Abdominal pain	Recto-sigmoid lesion	N/S	Defunctioning colostomy. Chemotherapy	N/A	7
	78 M	Curative resection	PDA	T3N3aM0	Abdominal pain and distension	Obstructing lesion in transverse colon		Resection of transverse colon	18	24
Lim *et al* 2011^9^	43 F	Curative resection	PDA + LP	T3N0M0	Constipation	Stricturing rectal lesion	Normal	Low anterior resection and defunctioning loop colostomy	34	Alive
R-Salaz *et al* 2010^10^	85 M	None. Synchronous metastases	SR	N/A	Routine colonoscopy	Gastric metastasis at colorectal anastomosis (previous sigmoid cancer)	Raised	None	N/A	12
Hamada *et al* 2019^11^	47 M	Curative resection	PDA	N/S	Routine colonoscopy	5mm rectal lesion	N/S	EMR	N/A	N/S
R-Diaz *et al* 2012^12^	58 M	Synchronous curative gastric and colonic resection	SR diffuse	N/S	Unclear	Multiple colonic lesions	N/S	Subtotal colectomy (synchronous with gastric surgery)	N/A	12
Tseng *et al* 2004^13^	74 M	Curative resection	N/S	N/S	Left lower abdominal pain	Multiple polypoid lesions in colon and rectum. All cystic septated and submucosal on EUS	N/S	Anterior resection	108	114
Tomikashi *et al* 2002^14^	57 M	None	PDA	N/A	Left flank pain, anorexia and weight loss	Multiple colonic submucosal polyps	Normal	Chemotherapy	N/A	N/S
Oh *et al* 2014^15^	67 F	Curative resection	PDA + SR	T2aN1M0	Haematochezia and weight loss	2 ulcerated fungating masses at 45cm and 25cm	N/S	Chemotherapy	36	N/S
Tanakaya *et al* 2004^16^	68 M	Curative resection	PDA + SR	T2N2M0	Lower abdominal pain	Ileocaecal lesion and stricture	Raised	Ileocaecal resection	96	112
Uemura *et al* 2016^5^	60 M	Curative resection after ESR ×2	WDA	N/S	Routine colonoscopy	Submucosal rectal mass	Raised	Laparoscopic low anterior resection	24	Alive
Tural *et al* 2012^17^	78 F	None. Synchronous metastases	WDA	N/A	Routine colonoscopy	Rectal mass at 10cm	Raised	Chemotherapy	N/A	Alive
Ogiwara *et al* 1994^18^	53 F	Curative resection	PDA	N/S	Weight loss, anaemia, melaena	10 left colonic submucosal polyps	N/S	None	132	Alive
Iwabuchi *et al* 2001^19^	74 M	None. Synchronous metastases	SR	N/A	Abdominal distension	Multiple gastric, colonic and small bowel lesions	Raised	Chemotherapy	N/A	Alive
Lee *et al* 2004^20^	41 M	None. Synchronous metastases	PDA + SR	N/A	Routine colonoscopy	Left colonic flat elevated polyps	Raised	Chemotherapy	N/A	1
Noji *et al* 2014^21^	61 M	Curative resection	PDA + SR	T3N0M0	Constipation, distension and pain	Stricturing left colonic tumour	N/S	Left hemicolectomy. Patient declined chemotherapy	108	Alive
	46 F	Curative resection	PDA + SR	T3N2M0	Constipation lower abdominal pain and distension	Rectal tumour invading uterus	N/S	Anterior resection with hysterectomy	108	Alive
Nakamura *et al* 2008^22^	86 F	None	PDA + SR	N/A	Epigastric pain	Non-polypoid flat elevated 5mm transverse colon lesion	N/S	EMR	N/A	9
Gao *et al* 2014^23^	81 M	Gastrectomy for benign disease many years prior	PDA + SR	N/S	Diarrhoea, weight loss, anorexia, abdominal pain	Multiple polyps throughout colon	Raised	Chemotherapy	N/A	3
Fujimoto *et al* 2016^24^	58 F	Curative resection	WDA + PDA + SR	T1bN0M0	PET CT on surveillance	Sigmoid lesion (and Krunkenberg left ovary)	Raised	Sigmoid colectomy (and oophorectomy). Chemotherapy	30	Alive
Pace *et al* 2009^25^	77 M	Curative resection	Mixed	T2bN2M0	PET CT on surveillance	Ascending colon tumour	Raised	Right hemicolectomy	12	N/S
Sonoda *et al* 2017^26^	81 M	Synchronous curative gastric and colonic resection	PDA + SR	T3N3M1 (peritoneum)	Routine colonoscopy	Transverse colon tumour	Raised	Transverse colectomy (synchronous with gastric surgery)	N/A	N/S
Katon *et al* 1989^27^	60 F	Exploratory laparotomy × 2	N/A	N/A	N/A	3 stricturing areas in colon	N/S	None	N/A	10
Then *et al* 2021^28^	66 M	Curative resection	N/S	N/S	Abdominal pain + weight loss	Apple core mass of mid transverse colon	N/S	Attempted stent, then en bloc resection of transverse colon mass	24	N/S

CT = computed tomography; EMR = endoscopic mucosal resection; ESR = endoscopic submucosal resection; EUS = endoscopic ultrasound; F = female; LP = linitis plastica; M = male; N/A = not applicable; N/S = not stated; PDA = poorly differentiated adenocarcinoma; PET = positron emission tomography; RIF =; SR = signet ring; WDA = well-differentiated adenocarcinoma

## Discussion

We describe two cases of patients who underwent curative intent gastric surgery for cancer presenting at 7 and 20 months postoperatively with rectal metastases. In both cases, the endoscopic appearances were not classical and the lesions in the rectum were felt to be submucosal, which in our first case led to misleading biopsy results.

The systematic review confirmed the assertion that metastasis of gastric cancer to the colorectum is rare; however, there are themes to be drawn from the 25 cases identified in the literature and summarised in Table 1.

### Histopathology

Perhaps unsurprisingly, the histopathology in the original resection specimens for our cases demonstrated poorly differentiated adenocarcinoma, which is associated with poor prognosis^29^ and an increased risk of lymph node^30^ and distant metastatic disease.^31^

In resected colonic specimens, it is notable that there was a distinct absence of any associated surrounding adenomatous polyps reported in the specimen and the lesions were located primarily in the serosal and submucosal layers of the bowel.^6^ This latter finding was replicated in our Case 1, with endoscopic biopsies being inconclusive and true diagnosis only being achieved after formal resection.

In an autopsy study of 331 gastric cancer patients, Verstegen *et al*^4^ found a significant difference in the histological features of gastric metastases to the colorectum favouring diffuse type; 1% of intestinal type cancers had metastases to the colorectum compared with 5.7% of diffuse type cancers (*p* < 0.05).

### Route of metastasis

Lymph node metastasis was common, both at the time of initial diagnosis of gastric cancer and the diagnosis of metastases;^6^ however, a single paper reported recurrence in the rectum in the absence of any lymph node metastasis either at the time of primary gastric cancer diagnosis or on presentation of the rectal metastasis. This case was treated with primary laparoscopic rectal resection with good oncological results.^9^

A review of 21 cases of late recurrence in the colorectum by Noji *et al* identified the transverse colon as the most common site of metastasis, suggesting a potential route of mesenteric spread.^21^ Indeed, one study demonstrates the route of metastasis of a gastric antral cancer along the para-aortic and middle colic lymph nodes to the transverse colon.^26^

Rectal metastasis of gastric antral cancer has been reported in the absence of hepatic or lymph node spread.^17^ In Case 2, there was strong histological evidence of a lymphovascular route of spread to the rectum.

### Endoscopic appearance of metastasis

The endoscopic appearance of colorectal lesions from gastric cancer metastases has been reported to be unique. Su *et al* comment on the wall thickening and swelling and the absence of ulceration in their two cases.^8^ In our first case report, the biopsies taken at the time of endoscopy were inconclusive and showed ulceration only. Similar difficulties obtaining histological diagnosis at endoscopy have been reported and one study describes how a ‘strip biopsy’ was required to get the diagnosis of malignancy.^16^ Submucosal lesions have been reported and can rarely be cystic in nature.^13^ It has been recommended by Tseng *et al* that endoscopic ultrasound is useful in the assessment of submucosal colorectal lesions.^13^

The usual appearance is of annular stricturing or linitis; however, multiple elevated flat polyps have also been described^20^ and depressed-type lesions have been reported.^19^ Ogiwara *et al* present a case with clustering of multiple polyps contacting metastatic gastric cancer,^18^ whereas another group ask us to consider clusters of colonic polyps as potential metastatic spread in patients with a known history of gastric cancer.^23^

### Diagnostic imaging

Lim *et al*^9^ recommend CT as the preferred modality for diagnosis because the appearance of intestinal metastasis from gastric cancer has been described previously.^32^ In our cases, the CT findings were not diagnostic, but MRI rectum showed unusual smooth anorectal thickening that was felt to be atypical for malignancy in Case 2.

Positron emission tomography (PET) CT is also recommended in the literature^7^ even when the tumours were not found to be PET avid.^6,7^ CT and barium enema findings can be unusual in colorectal metastases and findings can be confused with Crohn’s disease.^16,27^

### Tumour markers

Tumour markers including CEA, carbohydrate antigen 19-9 (CA19-9) and CA72-4 were elevated in 11 of the 15 papers that commented on tumour marker levels, making tumour markers an important part of both the diagnostic work-up and surveillance protocols in these patients.

### Management of colorectal metastases from gastric cancer

Management varies widely in the literature. For many, the presence of metastatic deposits of gastric origin in the colorectum signifies advanced disease so the treatment of choice was palliative chemotherapy or surgical resection only to alleviate symptoms.^7^ However, in some instances, in the absence of widespread peritoneal metastasis or other metastatic solid organ involvement, a more oncologically aggressive surgical approach was taken.^9,21,28^

One report by Wang *et al* describes an aggressive surgical approach even in the presence of peritoneal disease at the time of presentation of colonic metastasis. This group operated three times to remove disease in the colon, extending life expectancy after initial recurrence by 44 months.^6^

## Conclusions

Differential diagnosis in gastric cancer patients with new colorectal lesions, be they small and diminutive or large and stricturing, should include metastatic disease and the investigation and management should reflect that. Aggressive management of colorectal metastases in the absence of widespread metastatic disease and in a fit patient should be considered because this can prolong survival, but consideration must always be given to maintaining quality of life.
